# Video feedback intervention to enhance parental reflective functioning in primary caregivers of inpatient psychiatric children: protocol for a randomized feasibility trial

**DOI:** 10.1186/s13063-019-3310-y

**Published:** 2019-05-14

**Authors:** Fanny Leyton, Marcia Olhaberry, Rubén Alvarado, Graciela Rojas, Luis Alberto Dueñas, George Downing, Howard Steele

**Affiliations:** 1 School of Psychology, Pontificia Universidad Católica, Av. Vicuña Mackenna 4860, Macul, Santiago, Chile; 2 Department of Paediatrics. Faculty of Medicine, Universidad de Valparaíso. Subida Leopoldo Carvallo 200. Hospital Psiquiátrico del Salvador, Valparaíso, Chile; 3 Institut of Health Sciences, Universidad de O’Higgins, Rancagua, Chile; 4 Unit of Mental Health, School of Public Health, Faculty of Medicine, Universidad de Chile, Santiago, Chile; 5 Departamento de Psiquiatría y Salud Mental, Clínica Psiquiátrica Universitaria, Universidad de Chile, Santiago, Chile; 6 Salpetriere Hospital, Paris, France; 7 Psychology Department, New School for Social Research, New York, USA

**Keywords:** Video feedback intervention, Video intervention therapy, Parental reflective functioning, Inpatient psychiatric children

## Abstract

**Background:**

Children requiring hospitalization for psychiatric care have serious disorders, high use of psychotropic medication, and frequent readmissions. The development and implementation of therapies focused on incorporating primary caregivers or attachment figures is necessary for working with children with severe psychiatric disorders. Mentalization or parental reflective functioning (PRF) is the ability of parents to understand their children’s behaviors as an expression of internal emotional states and act accordingly to help them regulate their emotions; in this way mentalizing is a key component of sensitive parenting. Video-assisted therapies have proven to be effective in promoting change in parent–child relationships. The majority of studies have been carried out with mothers of pre-school children and in an outpatient setting. Video intervention therapy (VIT) is a flexible manualized therapy, which allows the intervention to be individualized to the context where it is applied, according to the needs and resources of the people who participate in it. The objective of the study is to evaluate the feasibility and acceptability of applying VIT to improve the PRF of the parents as primary carers of children hospitalized in a psychiatric service.

**Methods:**

This is a pilot randomized, single-masked (outcome assessor) study with a qualitative component. It will involve a block randomization procedure to generate a 2:1 allocation (with more people allocated to the intervention arm). The intervention consists of four modules; every module has both one video-recorded play session and one VIT session per week. People assigned to the control group will receive treatment as usual plus weekly play sessions. Feasibility and acceptability of the study will be quantitatively and qualitatively assessed. Evaluation of the caregivers will include assessments of PRF, wellbeing and personality structure; assessments of children will include parent-ratings and clinician-ratings of symptomatology and general functioning. After every video feedback (VF) session, PRF, the caregiver’s wellbeing and children’s general functioning will be reassessed.

**Discussion:**

This study will contribute to the currently scarce evidence on how to provide family attachment-based interventions in a child inpatient psychiatric unit. It will also inform the design and implementation of a future randomized clinical trial.

**Trial registration:**

ClinicalTrials.gov, NCT03374904. Registered on 14 December 2017 (retrospectively registered).

**Electronic supplementary material:**

The online version of this article (10.1186/s13063-019-3310-y) contains supplementary material, which is available to authorized users.

## Background

There is a growing need for inpatient hospital beds for young children requiring psychiatric care and a corresponding shortage of supply, with increased demand for hospitalization in the last decade [1–3]. In addition, hospital readmission is frequent and the disorders young children suffer are often severe, requiring high use of psychotropic agents [4, 5]. This paper describes the protocol of a feasibility trial with an intervention underway that aims to lessen the family burden of hospitalization of a young child, and to improve the quality of child-parent relationships.

It has been pointed out that compared with adolescents, younger children in psychiatric care tend to come from families with higher rates of psychosocial problems [6]. There is ample evidence relating family factors to the onset of psychopathologic conditions and poor outcomes in children, especially parental psychopathologic conditions [7–11]. Even if parents lose custody of their children during hospitalization or after discharge, most of them will continue having a relationship with them and maintain contact through visits [12], and hopefully these children will in time return to their families as better conditions are achieved. Knowing this reality, one of the challenges when a child is hospitalized concerns how best to work with the family. This task is complicated by hospital settings (the majority) in which the psychiatric unit is not equipped to provide a bed for a parent to stay together with his/her child in hospital. This optimal pattern of hospital care, where parent and child stay together, would facilitate the delivery of dyadic treatment or family therapy. But when parents are not in the hospital, the offer of some form of family intervention is often not taken up, and when treatment begins, there is poor adherence or retention [13].

In past decades, several attachment-based interventions have emerged, most often involving video-assisted therapy (see [14] where 15 of 21 chapters about attachment-based interventions concern early childhood interventions, the vast majority including video feedback (VF)). VF has been shown to be an especially powerful tool in promoting change in parent–child relationships, often in just a few sessions [15–20]. It seems that video helps parents to observe themselves from the outside and by replaying the video they can obtain a more realistic and adaptive perspective on the relationship they have with their children, and the direction in which they want to take the relationship [18].

To the extent that seeing oneself on video is an emotional experience, it is likely that the experience, in part, activates the attachment system, calling for emotion regulation skills [21] that a trained therapist can help the parent to achieve. Without adequate therapeutic support, parents who are shown a video of themselves with their children may feel alternately suspicious, fearful, shamed or exhausted.

This is why in the video-feedback intervention detailed below, which strongly relies on the approach of George Downing [12], therapists are trained never to judge a parent, and to highlight the parent’s strengths and, especially, those of the child. In this way, the parent’s and child’s nascent-emotion regulation skills, and the child’s capacity to explore, are praised and nurtured. An emphasis is placed on all the good things that are evident in the parent-child interaction, but in addition to that, the therapist asks the parent whether, given the opportunity to go back in time to the moment of interaction shown on the video, would they do anything differently. A consistent theme in the therapeutic work is to focus repeatedly on the child’s development and what can help the child become (more) school-ready and competent at peer relations.

The intervention also aims to promote sensitive parental behavior, understood to be based on the parent’s mentalization skills. Mentalization is defined as the capacity to understand and interpret one’s own behavior and that of others as an expression of mental states such as feelings, thoughts, fantasies, beliefs and desires [22]. This is based on research in parenting and child development that shows the importance of considering mental aspects underlying behavior in interactions between parents and children [22, 23]. Interactions with primary caregivers who are sensitive and attuned to their needs provide infants with a sense of being held in a safe environment [24], consistent with Bowlby’s definition of attachment [25]. Reflective functioning (RF) is the operational definition of mentalization and was initially validated as a measure mentalization in the context of an Adult Attachment Interview, which is highly correlated with child attachment at 12 months [14] (Fonagy P, Target M, Steele H, Steele M: Reflective-functioning manual version 5 for application to adult attachment interviews, unpublished).

Further evidence of the importance of mentalization or reflective functioning comes from studies showing that maternal sensitivity on its own is not enough to explain intergenerational transmission of secure attachment [26], whereas parental mentalization has been shown to fill this transmission gap [27–29]. Parental mentalizing is considered to have important implications for the development of self-regulation (Fonagy P, Target M, Steele H, Steele M: Reflective-functioning manual version 5 for application to adult attachment interviews, unpublished) [28, 30].

The majority of research in VF has been conducted with babies or toddlers although several authors have also described the use of VF techniques with older children such as preschoolers and adolescents [12, 31]. In a meta-analysis [15], only 6 of the 29 studies included children over 5 years old and the majority were small trials without control groups.

There is evidence that parental reflective functioning (PRF) relates to social adjustment and emotional regulation in preadolescents and adolescents [32, 33]. Therefore, improving PRF in the period of early childhood development could contribute to the promotion of better outcomes in young people. PRF might facilitate dialogue with children and foster a deeper understanding of their needs, thus contributing to their ability to face conflict and negative emotions appropriately [32]. In the context of children in psychiatric care, increasing their parents’ PRF might promote the quality of their relationships, improve treatment results, and prevent future hospitalizations.

Children in inpatient psychiatric care frequently come from multi-problem families that require specific, brief, and effective interventions. The intervention proposed in the current study is designed to respond to this need. A randomized feasibility trial was designed in which subjects were randomized to a psychotherapeutic intervention that used video-feedback to improve PRF, during the hospitalization of children and early adolescents admitted to a psychiatric unit. The comparison group, who will not receive VF, will receive typical care and play sessions.

Due to the scarcity of research into the use of reflective functioning (RF) with parents of hospitalized children with severe psychopathologic conditions, a feasibility study was designed as a first step to conducting a future effectiveness study. A pilot study can also identify key factors in the design and implementation of evidence-based interventions that need to be tailored specifically to the context of public health services. In this sense, a feasibility study would allow for the detection of specific strategies for the use of new therapeutic tools with parents and their children in hospital.

## Aims and objectives

The objective of this paper is to report on the protocol comprising a feasibility trial of VIT to enhance PRF in primary carers of children hospitalized in a psychiatric unit. As well as detailing the intervention, this paper provides an account of the plan to collect both quantitative and qualitative measurements of outcome.

## Methods/design

### Trial design

**Fig. 1:**
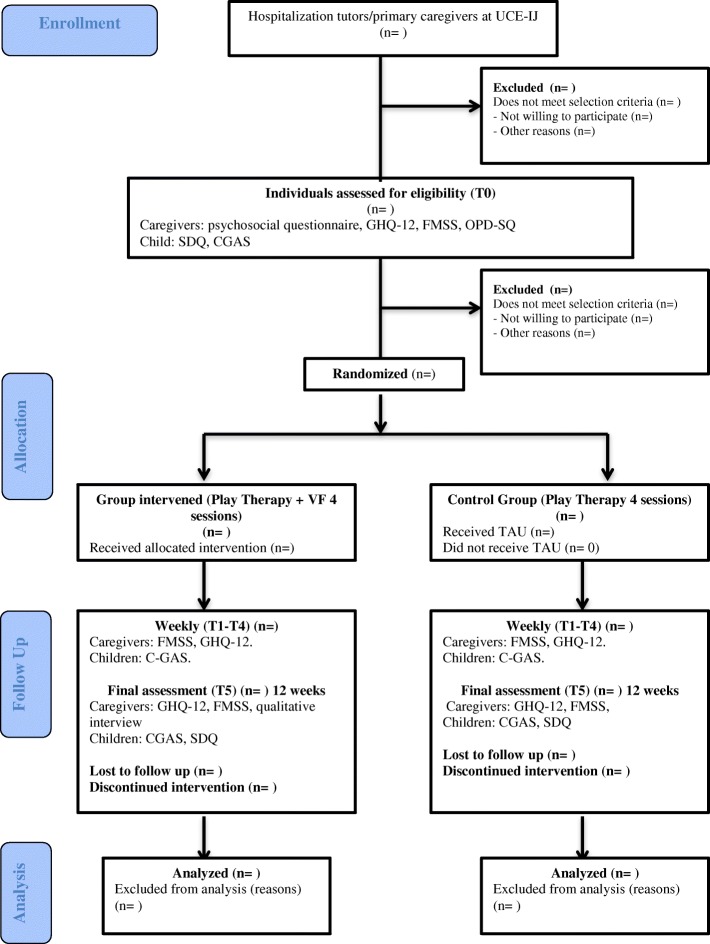
Flowchart of study phases and instrument application. TAU, treatment as usual; FMSS, Five Minutes Speech Sample; OPD-SQ, Operationalized Psychodynamic Diagnosis - Structured Questionnaire; GHQ, General Health Questionnaire; SDQ, Strengths and Difficulties Questionnaire; CGAS, Children Global Assessment Scale; VF, video feedback

A small randomized controlled feasibility trial with a qualitative component has been designed to assess the feasibility and acceptability of a brief VF intervention, and to collect parameters that may serve as the rationale for the implementation of a large randomized clinical trial (RCT) in the future. See Fig. 1.

### Settings and participants

The research will take place in a public child and adolescent psychiatry ward in Valparaíso, the Hospital Psiquiátrico del Salvador. The quantitative aspect of the study will be conducted with carers of children aged 6 to 14 years, who are hospitalized in a child and adolescent psychiatry ward from August 2017 until the complete sample size is attained, which is expected by December 2018. The sample will comprise all parents and/or caregivers who meet the inclusion criteria, do not meet the exclusion criteria, and who agree to participate. The expected sample size is 30 subjects in total; 10 in the control group and 20 in the experimental group. The 2:1 allocation was chosen to allow more data to be collected on the acceptability of the intervention to participants and the feasibility of delivering the intervention.

#### Inclusion criteria

The participant must be registered as a tutor during hospitalization or registered as the primary carer of the child or adolescent, and have legal or biological kinship with the hospitalized child or adolescent.

#### Exclusion criteria

Participants will be excluded if they are:

- Caregivers with severe intellectual deficit or psychotic symptoms
- Foster caregivers or institutional caregivers
- Parents who do not care for the child regularly (for example, they visit the child less than one week per month or have restraining orders)

For the qualitative component of the study, a minimum of six primary carers of children hospitalized in a psychiatric unit, who have participated in the VIT intervention will be interviewed, as well as three key stakeholders: a therapist, a nurse and the chief psychiatrist of the Special Care Unit.

### Power calculation

As a feasibility study, no hypotheses will be tested and, therefore, a formal power calculation is not appropriate [34, 35]. The unit where the research will take place has an average of 60 inpatient children per year, with almost half of these children in foster care and therefore with institutional caregivers who are not included in the study. Based on the feasibility of recruitment, we aim to have 30 participants in order to estimate key parameters for a future RCT to be based in large part on the primary outcome of the feasibility and acceptability trial, i.e. the caregivers’ reports of what was felt to be clinically useful or meaningful.

### Treatment

#### Control group (treatment as usual (TAU) + play therapy)

All patients and their families will be receiving standard care in a child and adolescent inpatient unit [36], which mainly focuses on the child’s individual symptoms and problems and includes pharmacological and daycare management, occupational therapy, crisis intervention and psychological counseling.

As all patients receive dyadic play therapy once a week in the company of their tutors, and only some of these pairs are invited to participate in VIT, dyadic play therapy will be the active comparator to VIT. Play sessions have a workshop format, where the caregiver plays freely with the child during each 45–60-min session. The type of play varies according to the child’s needs and developmental stage. There is a box of toys available for the children to explore, and they may participate in role play with their caregivers or play rule-based board games. Occasionally, young adolescents and their caregivers are invited to negotiate on a particular topic (e.g. time permitted for technology), plan a day off or think through what the routine on discharge will be. The type of play or the activities chosen are flexible, according to the child’s and caregiver’s particular needs as identified by the therapists [37]. Therefore, sessions consist of dyadic play interactions with tutors or other caregivers who are being coached by a therapist in promoting child-oriented and healthy social interactions. Five to ten minutes of these play sessions are video-recorded.

#### Intervention arm - video intervention therapy (VIT)

VIT is a technique for performing video feedback where behavior-oriented interventions and representational therapy elements are used [12, 18], providing a six-step video-analysis framework. Videos can be filmed at different settings, with the only requirement being an observable interaction of the child with his caregiver(s) where the full bodies and faces of all participants are ideally captured on the film [12, 20].

A four-module intervention was designed for this study. Each module includes a play session and a VIT session. First, a play interaction between the child and caregiver is recorded (5–10 min) during play therapy sessions. Then, the therapeutic team chooses chooses excerpts lasting approximately 1-2 minutes to display in VIT sessions. VIT occurs during the same week of play therapy and VIT excerpts are shown to groups of caregivers, unless there is only one study participant at that time. When VIT excerpts are shown in groups, caregivers view excerpts of multiple children, not just their own, and actively participate in the session. Interventions will be performed by the researcher and by a clinical child psychologist, both trained and supervised. The therapist prepares the feedback session to show positive interactions first. Then, if the caregiver is willing and psychologically prepared to explore problematic patterns that can be modified, the therapist further discusses these interactions with the caregiver. During the sessions, the therapist may shift focus based on real-time comments, questions and the group dynamic. The first session of VIT is centrally focused on building rapport with the caregiver, and reinforcing observed strengths of the caregiver, of the child, and of the caregiver-child relationships. The caregiver learns the immediate and longer-term developmental goals for the child from the therapist and other parents. Other caregivers or parents have a unique supportive role to play in VIT group sessions because of their peer status. Sometimes caregivers could spontaneously talk about something problematic that they would do differently if they were in that moment again, and sometimes therapists ask the parents if they want to see something that they could do differently (negative pattern); if the caregivers agree, they take a deeper look into the negative patterns using mentalization techniques. The cardinal virtue for the therapist of assuming a non-judgmental stance rests at the core of VIT work [12].

#### Procedure

Eligible participants will be caregivers of children in an inpatient unit. All caregivers who are referred to play therapy and meet inclusion criteria will be invited by a professional from the unit staff to participate in the VIT study, and they will be interviewed by one of the therapists to explain the study. Informed and written consent from caregivers and assent from children and adolescents participating in the study will be obtained before entry evaluation. The study includes the use of self-report questionnaires and samples of recorded caregiver monologues about their child, which are recorded in private, to assess parental reflective functioning.

#### Randomization and masking

An external researcher will use a random number generator to perform block randomization, then will create a list of participants before inclusion of the first participant, to provide allocation of 2:1 in order to have a higher number of participants in the VF intervention and to have a similar proportion of caregivers in both arms during the year. Only the main investigator is aware of blocking randomization. To avoid biases, the other members of the clinical team are in charge of the allocation of the caregivers. When a participant finishes the entry evaluation, the external researcher will inform the clinical team regarding the corresponding allocation.

Although participants and care providers will be aware of treatment allocation, encoders of PRF will be masked to this (outcome assessor masking). Transcriptions will be anonymous in order to mask the caregivers’ identities and whether they belong to the control or the intervention group. Three highly trained encoders, who are outside the therapeutic context, will analyze the interviews to ascertain the level of PRF.

### Outcomes

#### Feasibility parameters

The feasibility will be evaluated in terms of eligibility rates, recruitment rates and reasons for study refusals, data attrition and follow-up rates by treatment condition.

#### Acceptability of the intervention

Participant attendance rates, and caregivers’ and key stakeholders’ qualitative assessment of the acceptability of and satisfaction with the intervention. Acceptability will be evaluated in terms of attendance rates, and through a qualitative assesment from caregivers and key stakeholders of the intervention acceptability and satisfaction.

### Secondary outcomes

The secondary outcomes will be demographic and mental health status at baseline, change over time in PRF, caregiver’s wellbeing and children symptoms and general functioning.

### Instrument description

**Fig. 2:**
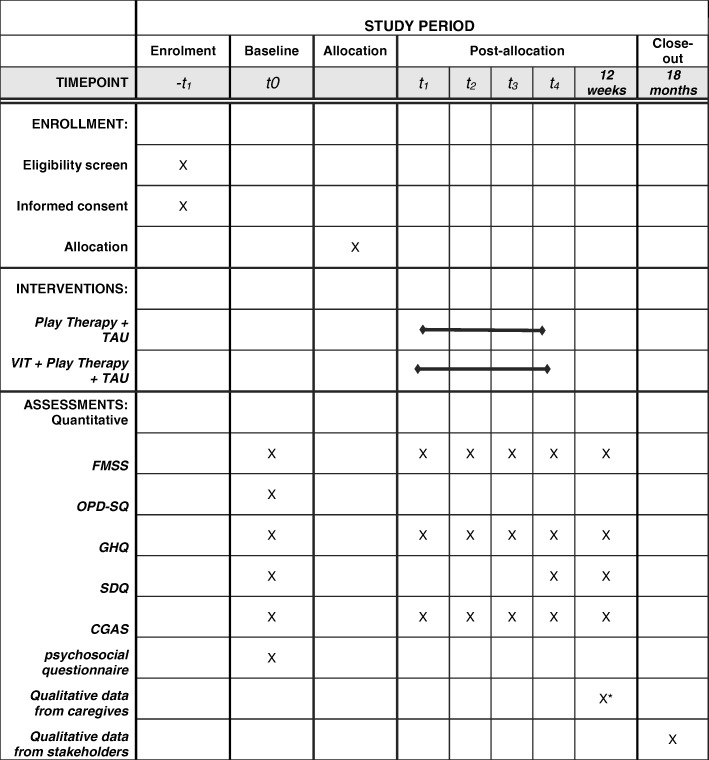
Schedule of enrollment, interventions and assessments. VIT, Video Intervention Therapy; TAU, treatment as usual; FMSS, Five Minutes Speech Sample; OPD-SQ, Operationalized Psychodynamic Diagnosis - Structured Questionnaire; GHQ, General Health Questionnaire; SDQ, Strengths and Difficulties Questionnaire; CGAS, Children Global Assessment Scale; VF, video feedback. *VIT participants only

Figure 2 shows the schedule of assessments. These are as follows:

1. Five Minute Speech Sample (FMSS) [38] for evaluating PRF
  In this instrument the caregiver is asked to talk about the child for 5 min without interruptions. This monologue is audio-recorded for future codification. The FMSS will be recorded for each caregiver at the beginning of the study and prior to each session. The FMSS has been used for over 30 years to assess the emotional expressiveness of parents towards their children [39], but over recent years its has increasingly been used as a tool for assessing parents’ or caregivers’ reflective functioning [40, 41]. RF levels are obtained by coding the transcription according to the *Reflective functioning evaluation manual* with a scale that goes from − 1 (avoidance or rejection of mentalization) to 9 points (complete or exceptional RF). A score of 5 indicates a clear understanding of mental states. The RF scale reliability after training is usually high, with correlation of 0.81–0.94 reported [32, 33, 42]. To date, there are no studies published in Chile that use the FMSS.
  The FMSS will be coded by a certificated psychologist with training in RF coding. To obtain inter-judge reliability in this sample, three coders will code 20% of the full set of FMSSs, i.e. 36 of the 180 to be collected [43]. The 36 FMSSs to be included in this test of inter-observer agreement will come more or less equally from each of the six assessment periods (six from each time period when PRF will be assessed).
  This tool will be applied upon entering, after each VIT session, and at the end of the study.
2. General Health Questionnaire [44] (GHQ-12)
  Araya et al. validated the GHQ-12 self-report questionnaire in Chile [45] and it is widely used there as a screening test for depression and general psychopathology [46]. In order to assess a person’s wellbeing, this instrument targets two areas: the inability to carry out normal functions and the appearance of distress [44], Total scores range from 0 to 36.
3. Operationalized Psychodynamic Diagnosis – Structured Questionnaire (OPD-SQ) [47, 48]
  The OPD-SQ self-report instrument measures the level of structural integration of personality through the evaluation of four main dimensions, which in turn can each be directed towards two orientations:
    i. Perception (of self and objects)
    ii. Management (of self and relations)
    iii. Emotional Communication (internal and external) and
    iv. Linkage (internal and external relationships).
  In each of its 95 items participants indicate on a 5-point Likert scale the degree to which they feel accurately described. The average of all items is an indicator of the global structural functioning, where higher scores indicate less structural integration. This instrument has been translated into Spanish and has been used amongst Chilean clinical populations [49].
4. Children Global Assessment Scale (CGAS) [47, 50]
  The CGAS is a clinician-rated tool used to assess general functioning in children aged 4–16 years. Scores range from 1 (the most impaired level) to 100 (superior functioning). Scores above 70 are considered to be near normal functioning [50]. This tool is commonly used by mental health clinicians in naturalistic settings and in research [51, 52]. Having been translated into Spanish, it is a valid and reliable scale both in time (intra-class correlation (ICC = .44)) and across evaluators (ICC = .81) [53].
5. Strengths and Difficulties Questionnaire (SDQ) [54]
  This self-report screening questionnaire assesses psychopathology in children and adolescents between the ages of 4 and 16 years. It can be completed by parents and/or teachers and takes approximately 5–10 min to complete. Each item is scored 0, 1 or 2 according to a Likert scale in three categories: not true, true and absolutely true. It also considers items that assess the child’s strengths, in which the scoring is inverted (0, absolutely true and 2, not true). This instrument has been validated in several countries showing good reliability. In Chile its psychometric properties have been evaluated in the parent population, showing good reliability in the total score and internal consistency with α = 0.79.
6. Sociodemographic survey
  A survey will be prepared according to the study’s aims, including individual and family data as registered upon patient entry to the Special Child Care Unit. Data will be collected on aspects such as age, parents’ educational level and employment status, children’s school achievements and failures and prior medical and/or psychiatric treatment, among others.
7. Participants’ interviews
  An open-ended set of questions will be given to caregivers at the end of the intervention. These questions include:
    i. What did they think was useful about the intervention?
    ii. What difficulties did they experience in engagement with the intervention?
    iii. Do they think their relationship with their child was changed by the intervention?
    iv. How did they experience the hospital treatment?
  These questions will be asked of the caregivers at the end of the intervention, to gain a picture of the acceptability of the intervention, and will be analyzed qualitatively.
8. Stakeholder interviews
  One of the therapists delivering the intervention will be interviewed with open-ended questions about aspects that need to be considered for the delivery of the intervention, such as time needed to prepare the session, how much supervision is required, etc. In addition, the chief psychiatrist and the unit charge nurse will be interviewed. Both will be asked about factors that they consider critical for implementing the intervention and what consequences in the functioning of the unit are observed during the development of the trial.

### Data collection

All participants will be assessed at baseline, immediately after every VIT session (after every play session for the control group) and 3 months after recruitment (Fig. 2). There is no economic compensation for participating in this trial.

### Safety monitoring and criteria for discontinuation

It is not predicted that there will be negative effects for the participants. Although any participant in a psychotherapeutic intervention might experience intense emotions, these will be addressed during the session. If any participants are identified through the questionnaires or clinical criteria as having mental health problems requiring a higher level of care, they will be referred to the corresponding health center. Participants may withdraw from the study at any time without any impact on the regular treatment their children are receiving on the inpatient unit.

### Data analyses

#### Qualitative study

The information obtained from the caregivers’ and key stakeholders’ interviews will be analyzed using grounded theory [55]. ATLAS.TI v7 software will be used for analyzing the data, as it enables managing and processing groups of text data.

#### Quantitative study

The quantitative study will be conducted as follows:

1. Descriptive statistics will be used for evaluation of the clinical and sociodemographic variables in the control and intervention groups. The mean and standard deviation (SD) will be calculated for continuous data and numbers and percentages will be calculated for categorical data.
2. Descriptive statistics will be used for evaluation of the eligibility and recruitment rate in the full sample; adherence, data attrition and follow-up rates will be calculated by treatment group.
3. The completion rate and missing data will be summarized for all variables.
4. Change over time in PRF, GHQ, SDQ and CGAS will be assessed graphically per group using a tangled line or spaghetti plot, displaying individual traces for each subject per group, and displaying the mean per group.
5. Estimates and variances of PRF, GHQ, SDQ and CGAS will be calculated to determine the most appropriate primary outcome measure for a definitive trial.

Data analysis and presentation of the results will be in accordance with Consolidated Standards of Reporting Trials (CONSORT) extension guidelines for randomized pilot and feasibility trials [35].

### Research governance and ethics

#### Trial management

The study will comply with local research governance requirements.

#### Ethics

Full ethical approval was obtained from the local Ethics Committee (Comité Ético Científico del Servicio de Salud Valparaíso-San Antonio, ORD 1502, 8 August 2017).

## Discussion

The study addresses an important gap in the knowledge on how to provide effective interventions for carers of children who are hospitalized in psychiatric units. As far as we know, children in need of inpatient psychiatric care come from multi-problem families in which most caregivers also suffer from mental disorders and in many cases do not receive any treatment [6, 9]. Considering this context, a brief, effective, attractive and low-cost intervention is required. Video-feedback interventions primarily focus on caregivers’ resources and strengths, facilitating the establishment of rapport with participants and promoting their attendance. Not being criticized and feeling they can effectively take care of their children, can be a new and attractive experience for them that promotes self-efficacy as a parent. The end-of-treatment interviews with parents will explore the range of reactions parent will have had to the intervention.

Although these interventions can be beneficial when working with parents of children in psychiatric care, not all evidence-based interventions can be easily implemented in public psychiatric health services, for different reasons. Clinical teams might resist modifying the type of interventions they are accustomed to using, due to lack of training, difficulties in accessing the necessary training or concerns about the usefulness of the intervention in naturalistic settings. For these reasons, the stakeholders are being interviewed.

This pilot study seeks to demonstrate that it is feasible to develop an innovative, manualized and potentially effective intervention for multi-problem families who have their children hospitalized in a public psychiatric service. This pilot trial will inform how to conduct a future trial in order to assess the effectiveness of VIT in improving PRF, psychiatric symptomatology in children and parent-child interactions. Likewise, future research in this area can explore further the relationship between PRF and child psychopathologic conditions, and the specific role that video feedback may play in promoting PRF [17] Additional file 1.

## Trial status

Recruitment of patients into the study began in August 2017. Recruitment ended in February 2019.

## Additional file

Additional file 1: SPIRIT checklist of protocol for a randomized feasibility trial: Video feedback intervention to enhance parental reflective functioning in primary caregivers of inpatient psychiatric children. (DOC 123 kb)

## Abbreviations

CGAS: Children’s Global Assessment Scale
FMSS: Five-minute speech sample
GHQ: General Health Questionnaire
OPD-SQ: Operationalized Psychodynamic Diagnosis – Structured Questionnaire
PRF: Parental reflective functioning
RCT: Randomized clinical trials
RF: Reflective functioning
SDQ: Strengths and Difficulties Questionnaire
TAU: Treatment as usual
VF: Video feedback
VIT: Video intervention therapy
